# Development of a three‐dimensional dose evaluation method for computed tomography

**DOI:** 10.1002/acm2.13897

**Published:** 2023-01-07

**Authors:** Toshizo Katsuda, Rumi Gotanda, Tatsuhiro Gotanda, Tadao Kuwano, Nobuyoshi Tanki, Kouichi Yabunaka

**Affiliations:** ^1^ Medical radiation technology Shizuoka College of Medicalcare Science Hamamatsu‐city Shizuoka Japan; ^2^ Faculty of Health Science and Technology Kawasaki University of Medical Welfare Okayama Japan; ^3^ Department of Radiology Osaka Center for Cancer and Cardiovascular Diseases Prevention Jyoto‐ku Osaka Japan; ^4^ Brain Activity Imaging Center ATR‐Promotions Inc Seika‐cho Kyoto Japan; ^5^ Department of Ultrasound Ono Memorial Hospital Nishiku Osaka Japan

**Keywords:** computed tomography, half‐cylindrical acrylic phantoms, radiochromic film, three‐dimensional dose distribution

## Abstract

During a single scan using computed tomography, an X‐ray tube orbits along a 360°‐circular path around the patient. A scan obtained using the half‐cylindrical type phantoms with a radiochromic film sandwiched in between reveals a pixel value map illustrating the two‐dimensional (2D) dose distribution. A three‐dimensional (3D) dose distribution can be obtained with a 360° rotation of the 2D dose map. This study evaluates the concept and methodology of creating a 3D dose map to develop a phantom with a radiochromic film for obtaining the 3D dose distribution. The coronal and axial plane dose distributions were also evaluated. A single scan computed tomography image obtained using a half‐cylindrical type of acrylic phantom with a sandwiched radiochromic film was studied. The diameters of the phantoms were 10 and 16 cm, and their lengths were 30 cm. A 2D image of the XR‐QA2 film was obtained using an image scanner and image processing software. A red channel image was used to obtain the 3D dose distribution using a computing platform. A pseudo color was applied to the red channel image from which cross‐sectional color images were obtained. Half of the cross‐sectional pixel data were rotated by 360° to generate the data for each axial plane. The image created was saved, and a 3D pixel value map was constructed. The dose measurement procedure for the 3D dose distribution was developed using half‐cylindrical acrylic phantoms with a radiochromic film.

## 1 INTRODUCTION

### 1.1 Importance of evaluating the radiation dose from computed tomography

Recently, radiation exposure during medical procedures has increased; particularly, due to the increased use of computed tomography (CT).^1^ Such exposure can increase the patients’ risk of developing cancer.^2^, ^3^ To mitigate radiation exposure during CT scans, an accurate estimation of the local and whole‐body radiation exposure is critical.^4^ Therefore, the dose to the entire exposed area must be determined. In this study, a three‐dimensional (3D) dose reconstruction method for CT was developed to determine the local doses and dose distributions.

### 1.2 Measurement method using an ion chamber

**FIGURE 1:**
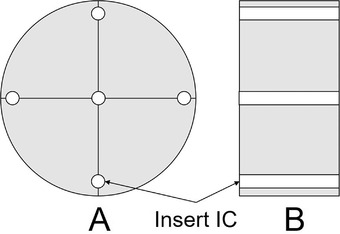
Diagram of the computed tomography phantom. A: Cross‐section. B: Long axis. IC: Pencil‐type ionization chamber. Four holes are present in the peripheral region, and one hole is present at the center of the phantom for IC insertion. The peripheral holes were located at a distance of 1 cm from the phantom surface. Therefore, the surface dose could not be measured. In addition, the X‐ray dose between the holes could not be measured

For the dose measurements, a 10‐cm‐long pencil‐type ionization chamber (10‐cm‐IC) and phantoms with diameters of 32 and 16 cm were used for the adult body and adult head, respectively.^5^ It is measured based on CT dose index (CTDI).^6^ The dose‐measurement positions and depths in the phantoms were fixed, as depicted in Figure 1.

### 1.3 **Problems in** CT **dose measurement with a 10‐cm‐IC (Poor spatial resolution)**

The radiation dose can be measured by inserting a 10‐cm‐IC into each hole of the phantom. This can provide measurements from two regions: the dose at the center of the phantom and the doses near the surface. However, the CT doses at the surface of the phantom and between the dose closer to the surface and the center of the phantom cannot be measured (Figure 1). Therefore, the spatial resolution in the axial plane is poor. In addition, the long axis of the phantom (the direction parallel to the rotation axis of the CT X‐ray tube) can be instantly evaluated using the 10‐cm‐IC. Unfortunately, the spatial resolution is poor because the 10‐cm‐IC only has a single measurement cavity. Consequently, an evaluation of the dose distribution using a 10‐cm‐IC is extremely difficult. Because, the CT dose index (CTDI) in ICRP87 gives only a rough index of patient exposure,^7^ it is not possible to isolate a 10 cm long cavity.

In addition, by measuring the center and near the surface of the phantom with a weighted sum of CTDI, the dose distribution in the phantom can be grasped to some extent.^8^

Hence, this apparatus cannot measure continuous dose changes. Therefore, it is not possible to estimate the dose distribution in the phantoms, and the maximum and minimum doses cannot be determined. Additionally, it is difficult to evaluate the local doses.

### 1.4 Gafchromic film

Gafchromic films are self‐developing dosimetry films that do not require processing or chemicals and can be handled under ambient room light.^9^, ^10^ The density of the active layer increases with the amount of exposure to X‐rays and ultraviolet rays.^11^, ^12^ Compared with that of the 10‐cm‐IC, the spatial resolution of radiochromic films is exceedingly high and thus measuring doses in local regions using radiochromic films is more convenient. However, as the density change is expressed using a pixel value and may differ for each batch of films, a calibration curve to convert the pixel values into doses must be created.

### 1.5 Phantoms for Gafchromic films

Two types of phantoms have been developed for CT dosimetry using Gafchromic films. Among these phantoms, one is a sheet role type of phantom in which a flexible acrylic sheet is wrapped around a cylindrical or oval core, and the Gafchromic film is sandwiched between the sheets for measurement.^13^ The other type is a half‐cylindrical phantom in which a half‐cylindrical solid acrylic pair is used, and a Gafchromic film is sandwiched between the pair for measurement.^14^

**FIGURE 2:**
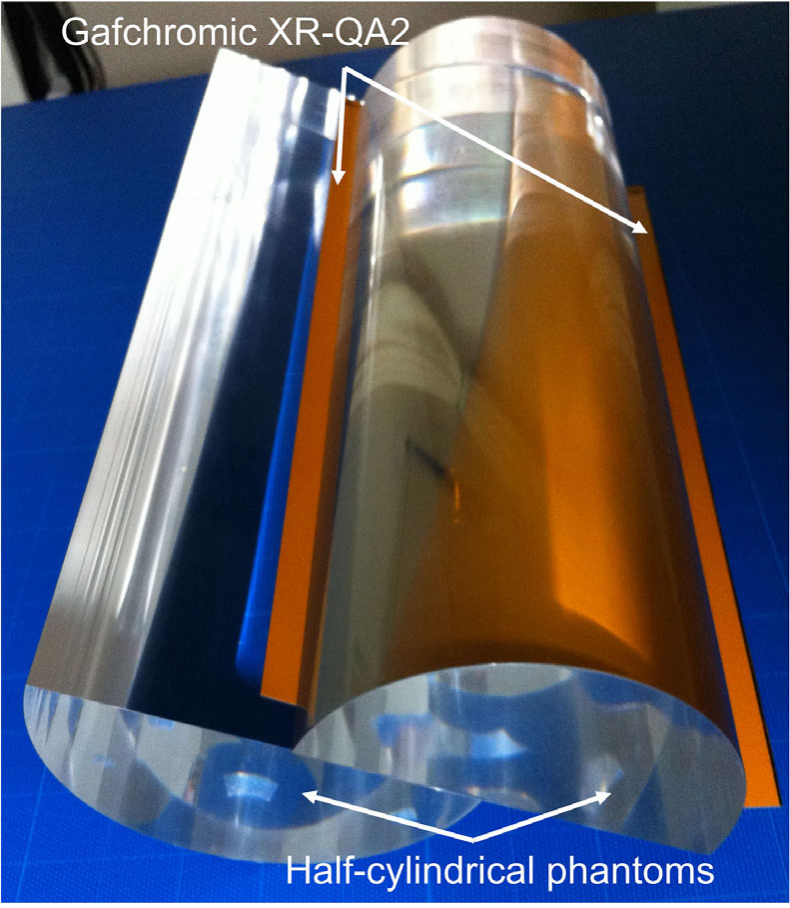
Half‐cylindrical acrylic phantoms with a Gafchromic RX‐QA2 film. Gafchromic RX‐QA2 film was sandwiched between the phantoms. As the scan data is inaccurate in the vicinity of the cut due to the presence of artifacts, the Gafchromic film was cut into pieces with dimensions larger than the diameter of each phantom. This was done so as to avoid recording inaccurate data

We attempted to use the half‐cylindrical phantom with a Gafchromic film to create the volume data of a CT scan (Figure 2).

### 1.6 Two‐dimensional and three‐dimensional data

When a single CT scan is performed using a half‐cylindrical acrylic phantom with a Gafchromic film, the measurable dose data are two‐dimensional (2D) data. However, when the entire exposure area is to be estimated, three‐dimensional (3D) volume data are required. The concept of creating 3D dose data has already been established in a previous study.^15^ This study demonstrates the evaluation of 3D data using axial images.

### 1.7 Purpose

The purpose of this study was to utilize a half‐cylindrical phantom with a Gafchromic film to obtain the dose information, such as the dose distribution evaluation by axial images and high‐resolution 3D dose distributions.

## 2 MATERIALS AND METHODS

### 2.1 Radiochromic films

Reflective‐type Gafchromic XR‐QA2 (XR‐QA2) (lot#: 07061601, Ashland, Inc., Covington, KY) was used as the radiochromic film in this study. The X‐ray sensitivity was 1–200 mGy. The corresponding energy was 20–200 kVp. The colors of the front and back surfaces were yellow and white, respectively. The film could be cut and handled under ambient lighting and required no cassettes or darkrooms. Moreover, it could be used underwater.^9^, ^10^

As the scan data is inaccurate in the vicinity of the cut due to the presence of artifacts, the Gafchromic film was cut into pieces with dimensions larger than the diameter of each phantom. This was done so as to avoid recording inaccurate data. The sizes of the XR‐QA2 pieces were 11 cm × 25 cm for the infant phantom and 17 cm × 25 cm for the adult phantom. The profile curves and volume data were created based on the diameter of the phantom.

### 2.2 Phantoms

Two half‐cylindrical phantoms made of acrylic material (WBI Co., Ltd. Neyagawa, Japan; Figure 2) were used in this study. The dimensions were 10 cm (infants) and 16 cm (adults) of the diameter and the length was 30 cm. The XR‐QA2 film was sandwiched between the phantoms. The phantoms were nonopaque; therefore, alignment of the scanning position was achieved using the laser systems of the CT.

### 2.3 Calibration curve

The XR‐QA2 film was divided into 14 segments, with each segment exposed to 2–200 mGy X‐rays. The exposure conditions were as follows: tube voltage of 80 kV, tube current of 200 mA, and exposure times of 0.032 s for 2 mGy, 0.08 s for 5 mGy, and 0.4 s for 25 mGy. The source‐to‐film distance was set to a base of 50 cm. A Thinx RAD semiconductor X‐ray analyzer (Unfors RaySafe AB, Billdal, Sweden) was used to measure the air kerma. The curve‐fitting equation was established using an IGOR Pro version 6.3.6J (Wave Metrics Inc. OR). The curve was fitted using “dblexp,” which is used to fit the sums of decaying exponentials.

### 2.4 CT scans

Single CT scans were performed using the 10‐cm‐ and 16‐cm‐diameter half‐cylindrical phantoms with the XR‐QA2 film. The scan conditions were identical to those of a conventional single scan: tube voltage of 120 kVp, tube current of 300 mA, and a 2.0 cm scan thickness. The scan time was 1 s/rot for the 10 cm phantom and 2 s/rot for the 16 cm phantom. The CT was performed using a clinical X‐ray CT (Aquilion Lightning, Canon Medical Systems, Ohtawara, Japan).

### 2.5 Image scan

The image scan of the XR‐QA2 was performed using an ES‐10000G (EPSON, Co., Ltd. Nagano, Japan) image scanner with the Adobe Photoshop CS2 (Adobe Systems Inc., San Jose, CA) in the red, green, and blue (RGB) channel mode at a special resolution of 150 dots per inch (DPI) and a density resolution of 48 bits. To reduce the Moiré artifacts, a liquid crystal display protection film (LCD‐230 W;Sanwa Supply Inc.;Okayama, Japan)^16^ was also utilized. To maintain consistency in the scanning position, the XR‐QA2 film was fixed on an acrylic plate with a thickness of 3 mm and was placed on the edge of the scanning area. The scans were performed after X‐ray irradiation to create a calibration curve and determine the pixel values.

### 2.6 Image analysis

To estimate the dose of the single‐scan CT image from the XR‐QA2, the color image was split into the RGB channels; the R color channel was used for image analysis with ImageJ (version 1.45i) for Windows (National Institutes of Health, Bethesda, MD).

### 2.7 Analysis of 2D data

**FIGURE 3:**
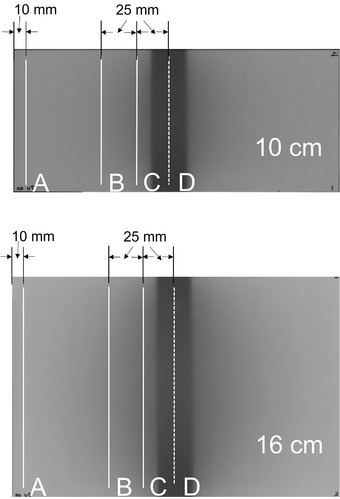
Regions of the Gafchromic XR‐QA2 film used to create the profile curves. The positions A, B, C, and D were used to create the axial plane pixel value maps. A: Profile curve along the peripheral region. B: Profile curve along the scattered region. C: Profile curve along the adjusted exposure region. D: Profile curve along the center of exposure region. As the scan data is inaccurate in the vicinity of the cut due to the presence of artifacts, the Gafchromic film was cut into pieces with dimensions larger than the diameter of each phantom. This was done so as to avoid recording inaccurate data. Therefore, the profile curve was not calculated based on the dimension of the Gafchromic film but based on the diameter of the phantom

The profile curves of the scanned XR‐QA2 film were created using five regions for both phantoms (Figure 3). Profile curves along the short axis were created for the peripheral region (A), scattered area (B), adjusted X‐ray exposure area (C), and center of the exposure region (D).

### 2.8 Principle of creating 3D volume dose data

**FIGURE 4:**
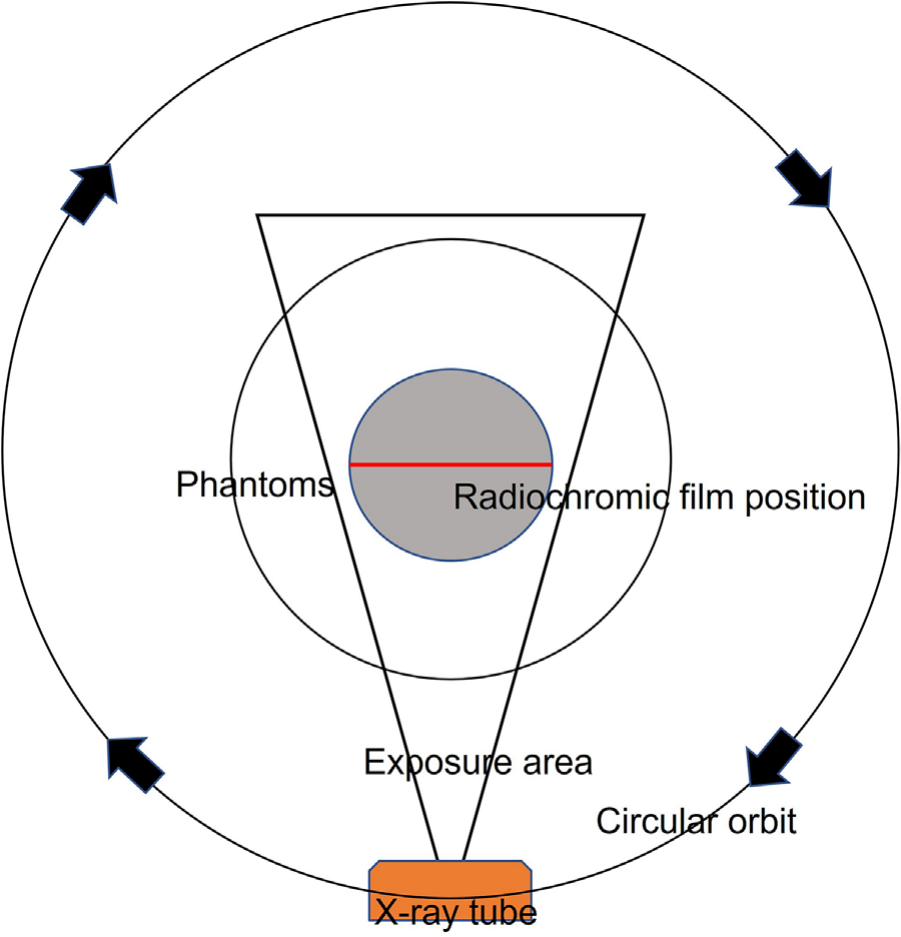
Principle of creating three‐dimensional volume dose data. The phantom with a Gafchromic XR‐QA2 film was placed at the center of the CT. A single scan was performed. The X‐ray tube was rotated around the phantom

**FIGURE 5:**
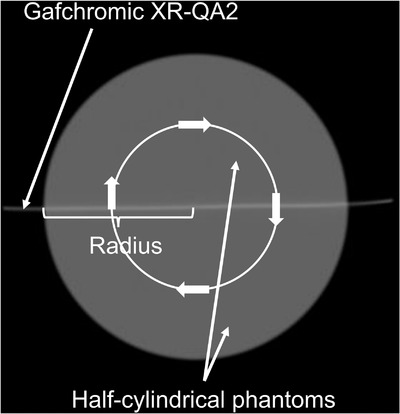
One of the actual CT axial images of the 10‐cm‐diameter phantom with a Gafchromic XR‐QA2 film. A Gafchromic XR‐QA2 film was sandwiched between half‐cylindrical acrylic phantoms. Both ends of the Gafchromic XR‐QA2 film protruded from the phantom. As the scan data is inaccurate in the vicinity of the cut due to the presence of artifacts, the Gafchromic film was cut into pieces with dimensions larger than the diameter of each phantom. This was done so as to avoid recording inaccurate data. Volume data can be created by rotating the data of the radius of the 360° (arrows)

A phantom with the XR‐QA2 film sandwiched in between was placed at the center of rotation of the CT, and the X‐ray tube was rotated by 360°. The XR‐QA2 film was irradiated with 360° X‐rays, and the 2D dose distribution through the center of the phantom was expressed as the density increase (Figures 4 and 5). Considering the center of the XR‐QA2 film as the center of rotation of the CT, the density data for the radius was chosen and rotated within the range of 360° to create the volume data. These data were used to create a cross‐sectional dose‐distribution map.

### 2.9 Analysis of 3D data

A grayscale image of the R channel was modified into a pseudo‐color image lookup table using the ImageJ software. The resolution was reduced to 50 DPI. The cross‐sectional dose distribution image was created by rotating the cross‐sectional radius data using MATLAB (MathWorks, Inc., MA) at positions similar to those in the 2D evaluation (Figure 3), such as the scattered area (B), the adjusted X‐ray exposure area (C), and the center of the exposure region (D). The created cross‐sectional images were saved in the Digital Imaging and Communications in Medicine format.

## 3 RESULTS

### 3.1 Calibration curves

**FIGURE 6:**
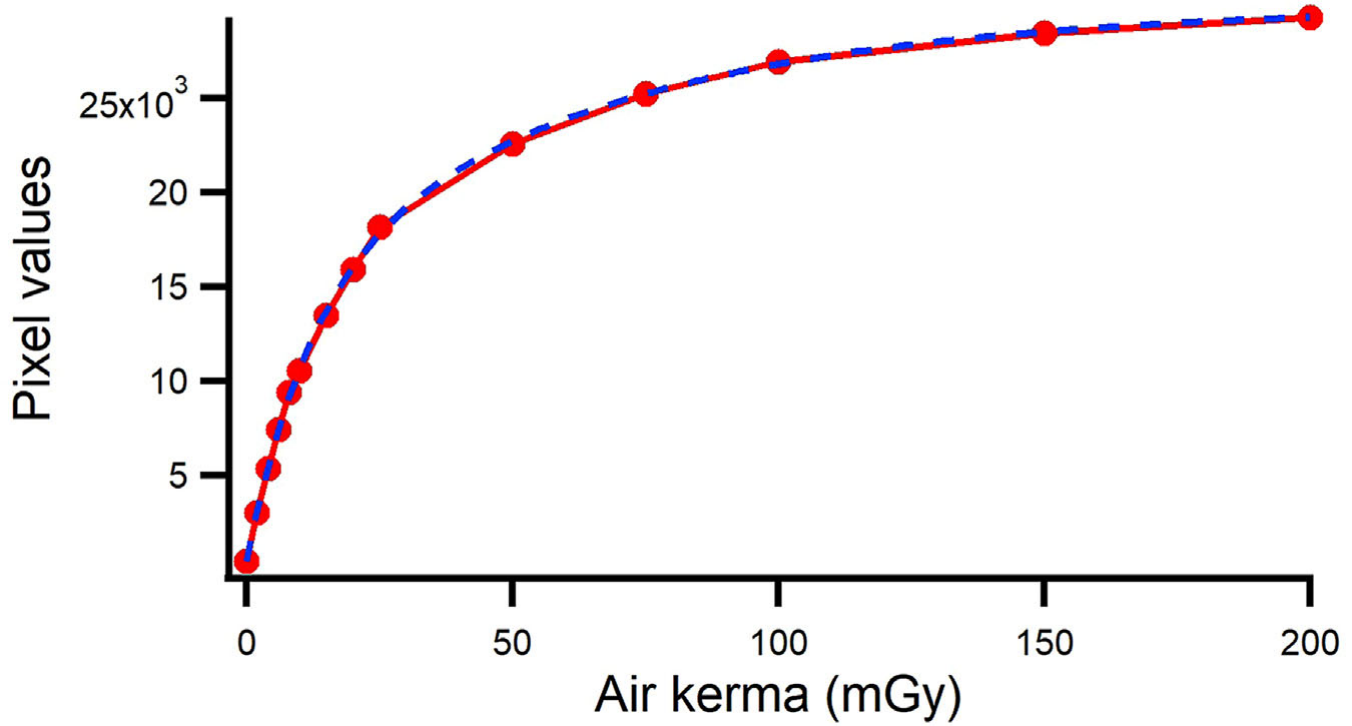
Gafchromic XR‐QA2 film for creating a calibration curve. The exposure method used is a segmented method. The exposure doses from 2 to 8 were 2.0 mGy, 10 and 20 were 5.0 mGy, and 25 to 200 were 25.0 mGy, respectively

**FIGURE 7:**
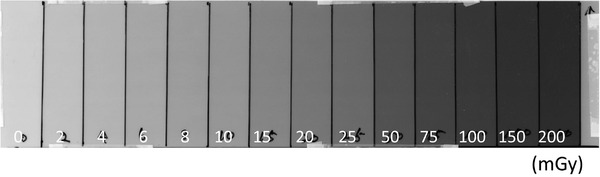
Calibration curve (dotted line with markers indicating measurement data) and fitted curve (solid line indicating sum of decaying exponentials)

The XR‐QA2 strip used to create the calibration curve is depicted in Figure 6, and the corresponding curve is illustrated in Figure 7. The raw data are listed in Table 1. The dose‐calibration curve was fitted using the following equation:

(1) $$\;y_{0}+\;A_{1}\exp\left(-B_{1}x\right)+A_{2}\exp\left(-B_{2}x\right)$$

**TABLE 1:** The relation between dose and pixel value

Dose (mGy)	Pixel values
0	0
2	2593
4	4914
6	7114
8	9047
10	10 328
15	13 295
20	15 672
25	17 911
50	22 275
75	24 957
100	26 567
150	28 078
200	28 944

The fitting curve coefficients were as follows:

$$y_{0}=29525\pm 323$$

$$A_{1}=-15404\pm 1.17e+003$$

$$B_{1}=0.01617\pm 0.0019$$

$$A_{2}=-14172\pm 1.35e+003$$

$$B_{2}=0.085367\pm 0.00771$$

### 3.2 Analysis of 2D data

**FIGURE 8:**
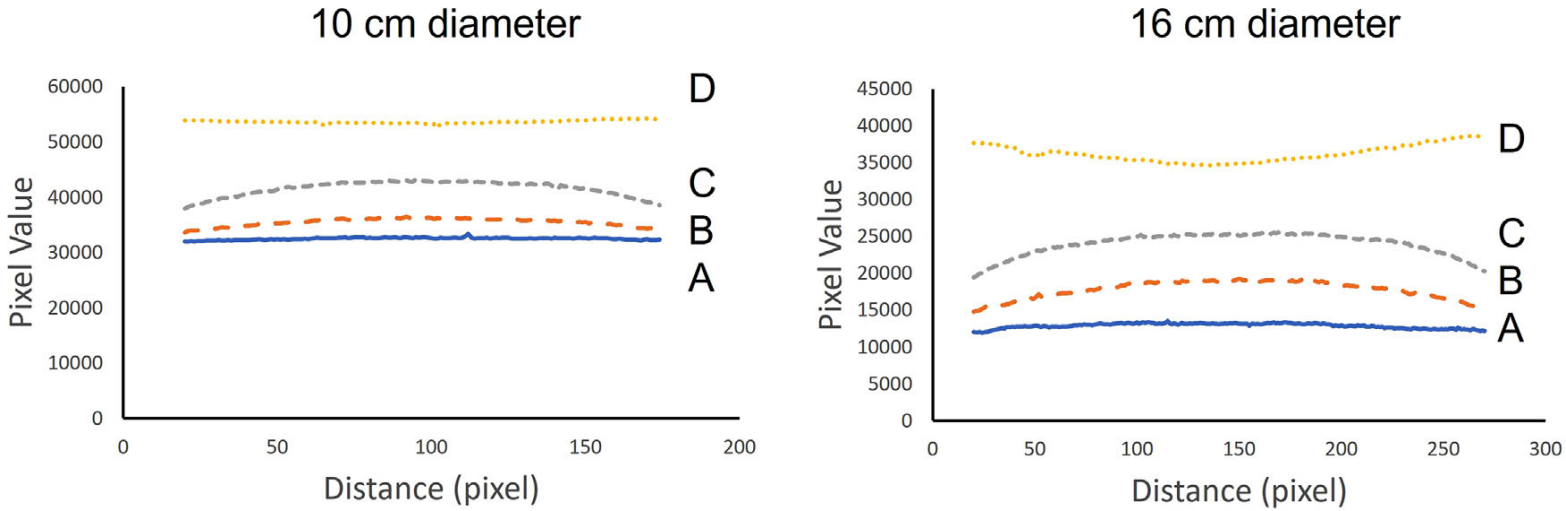
Four profile curves along the cross‐section of the phantom. The left side depicts the 10‐cm‐diameter phantom, and the right side depicts the 16‐cm‐diameter phantom. A: Profile curve along the peripheral region. B: Profile curve along the scattered area. C: Profile curve along the adjusted exposure area. D: Profile curve at the center of the exposure area. The profile curve was calculated using the diameter of the phantom. The X‐rays exposure region in the 10‐cm‐diameter phantom, the dose at surface regions were similar than that of central region (D). However, at scattered radiation area, the dose at surface regions were lower than that of central region (C, and B). The X‐rays exposure region in the 16‐cm‐diameter phantom, the dose at surface regions were higher than that of central region (D). However, at scattered radiation area, the dose at surface regions were lower than that of central region (C, and B)

The profile curves along the short axis for the exposure and scattered X‐ray regions of the 2D XR‐QA2 film are depicted in Figure 8. The profile curves for the peripheral regions of the X‐ray exposure area in both phantoms were almost flat (A in Figure 8). In contrast, the profile curves for the adjusted X‐ray exposure area and scattered areas exhibited higher pixel values in the central regions than those in the peripheral regions (B and C in Figure 8). For the profile curve at the center of the exposure area, the pixel value in the central region was slightly lower than that in the peripheral region of the infant phantom (D in Figure 8, left). However, for the adult phantom, the pixel values in the central region were significantly lower than those in the peripheral regions (D in Figure 8, right).

### 3.3 Analysis of 3D data

**FIGURE 9:**
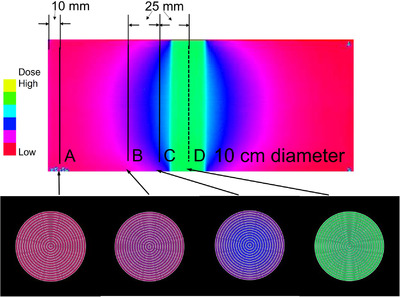
Four axial plane images of the 10‐cm‐diameter (infant) phantom. Lines A to D indicate the positions for calculating the axis data. A: The peripheral region. B: The scattered area. C: The adjusted exposure area. D: The center of exposure area. The X‐rays exposure region in the 10‐cm‐diameter phantom, the dose at surface regions were similar than that of central region (D). However, at scattered radiation area, the dose at surface regions were lower than that of central region (C, and B)

**FIGURE 10:**
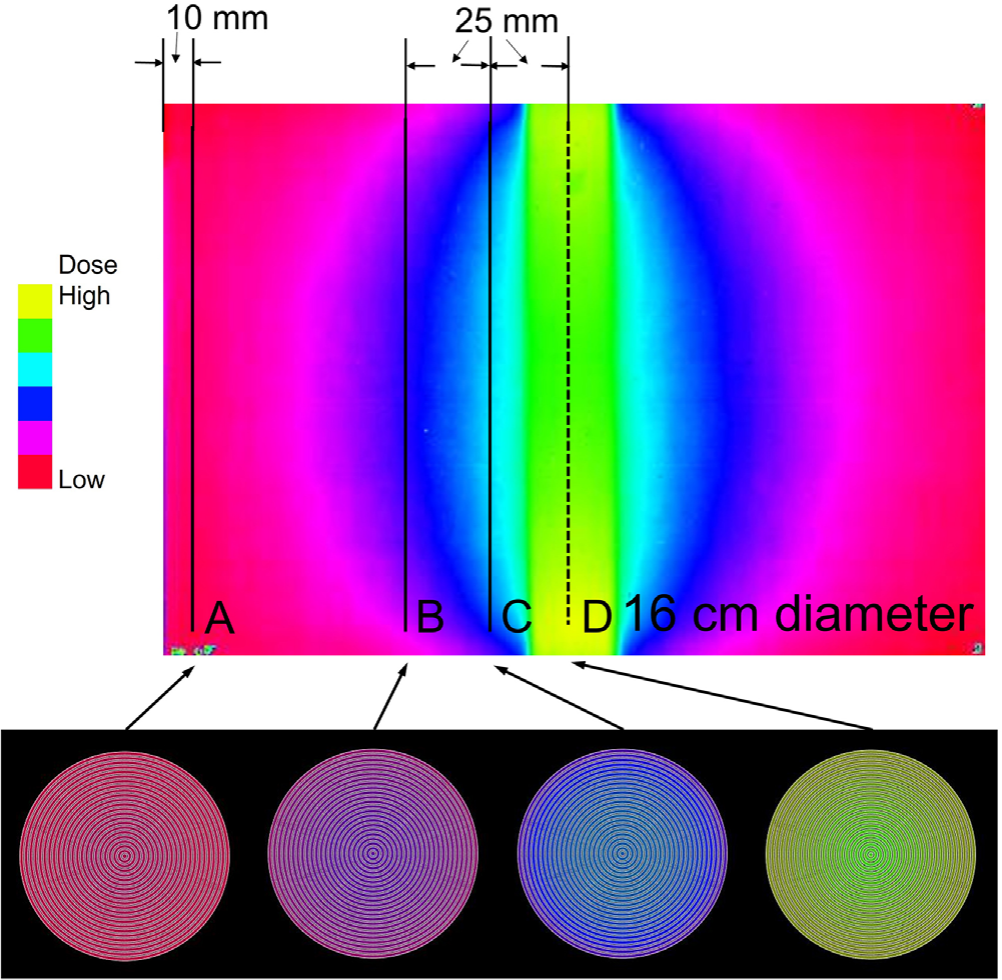
Four axial plane images of the 16‐cm‐diameter (adult) phantom. Lines A to D indicate the positions for calculating the axis data. A: The peripheral region. B: The scattered area. C: The adjusted exposure area. D: The center of exposure area. The X‐rays exposure region in the 16‐cm‐diameter phantom, the dose at surface regions were higher than that of central region (D). However, at scattered radiation area, the dose at surface regions were lower than that of central region (C, and B)

A pseudo‐color image of the XR‐QA2 film for the child phantom is presented at the top of Figure 9. The constructed cross‐sectional images for the four regions are illustrated at the bottom of Figure 9. The images created for the adult phantom are illustrated in Figure 10.

In Figure 9 (infant), the green region at the center represents the X‐ray exposure area, while the blue region represents the adjusted and scattered areas. The red region denotes the area that was not exposed to X‐rays. From left to right, the cross‐sectional images depict the peripheral region (A), scattered radiation region (B), adjusted region (C), and X‐ray exposure region (D). In the scattered radiation and boundary regions, the colors differ between the center and the peripheral region, and the strength and weakness of the scattered radiation are depicted.

In Figure 10 (adult), the yellow and green regions at the center represents the X‐ray exposure area, while the blue region represents the adjusted and scattered areas. The red region denotes the area that was not exposed to X‐rays. From left to right, the cross‐sectional images depict the peripheral region (A), scattered radiation region (B), adjusted region (C), and X‐ray exposure region (D). In the scattered radiation and boundary regions, the colors differ between the center and the peripheral region, and the strength and weakness of the scattered radiation are depicted.

For the X‐ray exposure area in the child phantom, as the color variation between the center and the edge is minimal, we can assume that the center dose was similar to the peripheral dose. However, in the adult phantom, the center dose was lower than the peripheral dose.

## 4 DISCUSSION

### 4.1 Scans

#### 4.1.1 Single scan

In this study, a method for generating the 3D dose data from the 2D dose data using a radiochromic film was evaluated. Specifically, a single CT scan in which the X‐ray tube is rotated through 360° around the patient was investigated. The single scan also included a cone‐beam CT with a thicker beam slice. By constructing the 3D dose data, it was possible to understand the dose distribution in the phantom. In particular, the dose distribution in the scattered radiation region could be established. Since it can be easily measured, changes in the dose distribution can be confirmed by changing imaging conditions such as tube voltage, tube current, and imaging time, which helps to optimize the dose.

In the future, it is necessary to determine how the 3D dose data can be generated from the 2D dose data for helical CT scanning.

#### 4.1.2 Under‐scan and over‐scan

The proposed method is valid only when the X‐ray tube rotates through 360°. In the case of an under‐scan (where the rotation is less than 360°) or an over‐scan (more than 360°), it is necessary to adopt a method that considers the rotation angle of the X‐ray tube using the image of the radiochromic film. The 2D data must be analyzed to generate the 3D data while accounting for the rotation of the X‐ray tube.

#### 4.1.3 Phantom position and phantom shapes

If the center of the half‐cylindrical phantom used in this study deviates from the center of the CT tube rotation, the 2D dose distribution can be evaluated. However, this method is not employed in the generation of 3D data as the radius data are not symmetric. Although not easy, there is also a sheet roll phantom^17^ method that uses a flexible acrylic with radiochromic films.

Similarly, this method cannot be used when the shape is not circular shape but elliptical shape. In this case, it is possible to measure 3D dose distribution by using a sheet roll phantom^13^ with a radiochromic film and making the shape of the winding core oval.

#### 4.1.4 Calibration curves

Density‐dose calibration curves for radiochromic films cannot be created using the CT and 10‐cm‐IC. Therefore, other X‐ray equipment and dosimeters must be used. The method for plotting the calibration curve for the density‐dose conversion will be described in another study.^18^ In the present study, an image evaluation method for the 2D data and a method for generating the 3D data from the 2D data are proposed, and the usefulness and feasibility of these methods are evaluated.

### 4.2 Evaluation

#### 4.2.1 Pixel value

In the 2D evaluation, the differences in the profile curves were evaluated based on the pixel values. The results indicated that it is possible to determine the local dose and dose distribution, which cannot be determined when using a 10‐cm‐IC. The profile curves presented herein present the density, distribution, and continuity of the change in the target area.

When using a 10‐cm‐IC, the dose along the long axis of the phantom could not be determined. Additionally, local doses and dose distributions could not be evaluated, as the number of ions received by the entire dosimeter were combined with a poor spatial resolution. Furthermore, as the dose data at the center and the peripheral regions of the phantom were added across the long axis direction of the phantom, the local doses and dose distribution could not be evaluated.

The 2D data presented herein reveal the difference between the generic dose obtained by a 10‐cm‐IC and the more specific dose distributions obtainable using a radiochromic film.

#### 4.2.2 Color images for 3D doses

For evaluation of the 3D dose distributions, color changes were observed in the pseudo‐color image. The color change in the short‐axis cross‐sectional image depicted at the bottom of Figures 9 and 10 indicates the dose differences. By connecting the dose information along the cross section of the phantom in the long‐axis direction, the local dose at any point in the phantom can be evaluated, and the continuity and distribution of the dose change can be determined. Although the X‐ray dose distribution in the exposure area appears to be uniform upon examination explicitly based on the color information, it was originally possible to evaluate this using the 2D X‐ray dose. By optimizing the dynamic range setting, it is possible to evaluate the dose differences in the exposure area.

#### 4.2.3 Dose distribution

Although it depends on the diameter of the phantom, the X‐ray dose distribution on the cross‐section of the phantom in the scan region was found to be lower in the center than in the peripheral region. This difference was observed to increase with the phantom diameter.

However, for the scattered radiation region, the dose distribution at the center was observed to be higher than that in the peripheral region for both the 10 and 16 cm phantoms. This is because the primary X‐rays were incident on the surface of the phantom, whereby they were absorbed from the peripheral area toward the center; thus, the distribution was lowest on the far side from the incident point. For the scattered X‐ray region, lateral scattering from the incident X‐rays increased the scattered X‐rays in the central part of the phantom, where the affected region was the largest. This dose information could not be obtained using the 10‐cm‐IC.

## 5 CONCLUSION

In this study, a method for converting the single‐scan CT dose data from 2D to 3D was proposed. A simple program was developed to achieve this conversion. In a high‐resolution dose measurement, the X‐ray exposure region and scattered X‐ray region were separated, and local doses and dose distributions were evaluated, which was not possible from the dose measurements obtained using a 10‐cm‐IC.

## AUTHOR CONTRIBUTIONS

Toshizo Katsuda: All related research and writing of the paper. Rumi Gotanda: X‐ray irradiation on CT and related research. Tatsuhiro Gotanda: Creation of calibration curve and related research. Tadao Kuwano: Gafchromic film imaging and related research. Nobuyoshi Tanki: X‐ray irradiation on CT and related research. Kouichi Yabunaka: Data analysis, curve fitting and related research.

## CONFLICT OF INTEREST

There are no conflicts of interest regarding this work.
